# One-Step Purification of Microbially Produced Hydrophobic Terpenes via Process Chromatography

**DOI:** 10.3389/fbioe.2019.00185

**Published:** 2019-07-29

**Authors:** Ljubomir Grozdev, Johann Kaiser, Sonja Berensmeier

**Affiliations:** Bioseparation Engineering Group, Department of Mechanical Engineering, Technical University of Munich, Garching, Germany

**Keywords:** terpenes, preparative chromatography, purification, fermentation, process development and integration, β-caryophyllene

## Abstract

Novel and existing terpenes are already being produced by genetically modified microorganisms, leading to new process challenges for the isolation and purification of these terpenes. Here, eight different chromatographic resins were characterized for the packed bed adsorption of the model terpene β-caryophyllene, showing their applicability on an *Escherichia coli* fermentation mixture. The polystyrenic Rensa^®^ RP (Ø 50 μm) shows the highest affinity, with a maximum capacity of >100 g L^−1^ and the best efficiency, with a height equivalent of a theoretical plate (HETP) of 0.022 cm. With this material, an optimized adsorption-based purification of β-caryophyllene from a fermentation mixture was developed, with the green solvent ethanol for desorption. A final yield of >80% and a purity of >99% were reached after only one process step with a total productivity of 0.83 g h^−1^ L^−1^. The product solution was loaded with a volume ratio (feed to column) of >500 and the adapted gradient elution yielded a 40 times higher concentration of β-caryophyllene. The adsorption-based chromatography represents therefore a serious alternative to the liquid-liquid extraction and achieves desired purities without the utilization of hazardous solvents.

## Introduction

Terpenes build the largest group of natural products, many of them with a proven and relevant bioactivity, resulting in central substances in medicine (Howat et al., 2014). A general transformation from classical synthesis routes (extraction from plant material or chemical synthesis) toward a sustainable biotechnological production or biotransformation is taking place (Chang and Keasling, 2006; Frense, 2007; Bhatti and Khera, 2013; Ongley et al., 2013; Song et al., 2014; Awan et al., 2016). Important developments have been made in the field of metabolically engineered microbes to produce potent and valuable terpenes (Martin et al., 2003; Keasling et al., 2012; Yamada et al., 2014; Li et al., 2015; Leavell et al., 2016; Dziggel et al., 2017). The major anticancer agent Taxol^®^ is produced in plant cell suspension cultures (Choi et al., 2001; Jennewein and Croteau, 2001; Malik et al., 2011) and offers further possibilities for biosynthesis (Guerra-Bubb et al., 2012; Jiang et al., 2012; Liu et al., 2016), which also includes its derivatives (Rohena and Mooberry, 2014). A Taxol^®^ precursor, Taxa-4,11-diene, can also be synthesized in a recombinant *Escherichia coli* (*E. coli)* strain (Huang et al., 2001; Ajikumar et al., 2010; Boghigian et al., 2012; Soliman and Tang, 2015; Biggs et al., 2016; Ward et al., 2018). Both precursors of the anti-malaria agent artemisinin, amorpha-4,11-diene and artemisinic acid, can be synthesized on a technical scale with a yeast and *E. coli* (Newman et al., 2006; Ro et al., 2006; Tsuruta et al., 2009; Paddon et al., 2013). These active compounds must reach an extremely high purity (>99%) for their application in medicine, which is generally achieved through multiple process steps after the fermentation. Nevertheless, challenges for the purification are given by the low concentrations of the products and the high number of impurities, such as host cell proteins (HCP) and media components.

Current purification strategies for fermentatively produced terpenes are limited to extractions with organic solvents (Janoschek et al., 2018). The processes for other hydrophobic natural products are developed empirically with non-optimal conditions and standard unit operations, which derive from the classical natural product isolation (Kawasaki et al., 2008; Sreekanth et al., 2009; Sun et al., 2009; McPartland et al., 2012; Bergs et al., 2013; Silva et al., 2018b). Chromatographic processes are often preferable and unavoidable, when purity requirements must be met, and can be developed specifically for the product binding and desorption, leading to extremely high purities after just one unit operation (Xiong et al., 2019). The adsorption of hydrophobic natural products, either in a packed (Wen et al., 2015; Saffarionpour et al., 2018a) or in an expanded bed (Gailliot et al., 1990), is an alternative to the classical extraction. Winkelnkemper investigated a theoretical approach for the purification of biotechnologically produced terpenes and especially 10-Deacetylbaccatin III, a Taxol^®^ precursor, where the isolation on a packed bed from a plant extract was demonstrated (Winkelnkemper and Schembecker, 2010; Winkelnkemper et al., 2011). Further work by Silva et al. (2018a) and Braga et al. (2018) showed a great potential for the packed bed chromatography to purify polyphenols after microbial production. Another advantage of a chromatographic purification over an extraction is the reduction of hazardous organic solvents (Schmidt-Traub et al., 2013).

In general, the first and most crucial step in process chromatography is the selection of the best suited stationary and mobile phase, with the highest possible separation factor and a high solubility of the product in the mobile phase (Felinger and Guiochon, 1998; Guiochon, 2002). Many parameters of the adsorbent can be considered and influence the performance, such as the chemical structure, the grade of crosslinking and the particle properties. In an adsorption-based chromatography with further elution of the product, the solvent for elution must be chosen properly for an optimized and cost-effective step. Hydrophobic natural products are easily separated by hydrophobic resins and then eluted with organic solvents (Krings et al., 1993; Schmidt-Traub et al., 2013; Silva et al., 2018a). Appropriate adsorbents exist in a wide variety starting from small particle sizes for analytical and semi-preparative purposes and going to large particles with broad distribution and low cost. Their chemical structure can be distinguished between polymers, such as polystyrene and inorganic materials with hydrophobic functionalization, such as silica with an aliphatic hydrocarbon. Polymers can be additionally functionalized and have different degrees of crosslinking. This variety leads to an immense number of possible adsorbents for one process. The cost of the downstream processing for small molecules produced by fermentation lies in a range of 20–40% in comparison to the total production cost (Straathof, 2011). Aside from the cost constraint given by the value of the product, the process parameters, such as the efficiency of separation and maximum productivity must be additionally considered (Guiochon, 2002).

The main properties of adsorbents needed for terpenes are a high selectivity, a high capacity and a quick and complete recovery by elution. Process properties include low specific backpressure and high column efficiency regarding a low height equivalent for a theoretical plate (HETP). This is mostly important for the gradient elution, where the bed efficiency influences the selectivity, purity, and concentration of the product. Here, smaller particle sizes can deliver a possible advantage in overall process economics.

The aim of this work is to characterize systematically eight different materials for the purification of the model terpene β-caryophyllene according to the above-mentioned criteria. This sesquiterpene is extremely hydrophobic (LogP_octanol/water_ > 5) and is a suitable substitute for further hydrophobic terpenes, which are non-volatile. The here applied methods and approach in the process development can be transferred for the chromatographic characterization of other fermentatively produced terpenes. By that, the best suited resin was implemented for the efficient purification of β-caryophyllene from a real *E. coli* fermentation mixture.

## Materials and Methods

### Adsorbents and β-Caryophyllene

**Figure 1:**
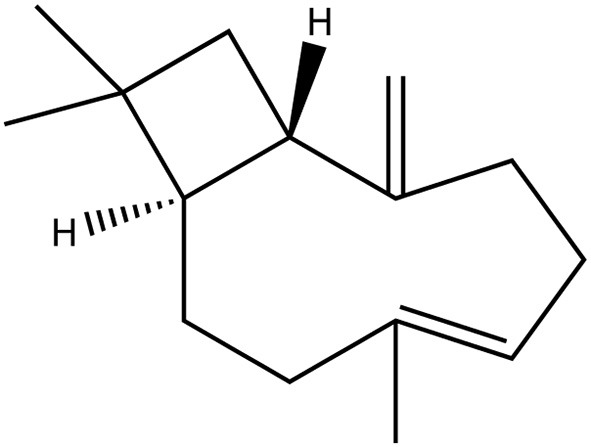
Chemical structure of the experimentally investigated sesquiterpene β-caryophyllene, consisting solely of hydrocarbons. It is extremely hydrophobic and poorly soluble in water with an octanol-water partition coefficient logP > 5.

The particle sizes vary from 40 to 1,000 μm, the specific surface area from 10 to 550 m^2^ g^−1^ and the cost per weight from 111 to 18,600 € kg^−1^. Adsorbents were chosen based on their potential for terpene purification. Those include mostly hydrophobic polymeric or inorganic materials with possible functionalizations (see Table 1). The Bio-Beads™ S-X12 and SM-2 were both sponsored by Bio-Rad Laboratories, Germany. The former is a polymeric size exclusion material, which gives defined porosities in a swollen state. Polygoprep™ C18 was purchased from Macherey Nagel, Germany. Rensa^®^ PY and RP were purchased from Biotage, Sweden. Silica gel 60, Amberlite^®^ XAD-2 and XAD-7 from Sigma-Aldrich, USA. The dimensions of the chromatographic glass columns (Omnifit^®^) were 0.66 cm and 1 cm in diameter, with a variable height from 0 to 15 cm. Frits with a mean pore size of 10 μm and made of PTFE were used. Ethanol was >99% pure, acetonitrile >98% and both were purchased from VWR, Germany. β-caryophyllene (CAS-Nr. 87-44-5, see Figure 1) was purchased from Molekula GmbH with a purity of >98%.

**Table 1:** Overview of the adsorbents for capturing the terpene β-caryophyllene.

**Adsorbent**	**Chemical structure**	**(Mean) particle size, μm**	**Specific surface area, m^**2**^ g^**−1**^**	**Mean pore size, Å**	**Cost, € kg^**−1**^**
SM-2	PS-DVB	200–1000^b^	286^a^	90	5,000
S-X12	PS-DVB	40–80	10^a^	–	7,800
Polygoprep C18	Silica + C18	63–200	350	60	1,107
Rensa^®^ PY	PS-DVB + Pyridin	100	500	50	18,600
Rensa^®^ RP	PS-DVB	50	320	120	6,200
Silica gel 60	Silica	40–63	450–550	55–65	111
XAD-2	PS-DVB	250–841	300	90	324
XAD-7	Polyacrylate	250–841	380	300–400	169

*Chemical Structure, particle properties, and cost per weight are listed. All except for one (Silica gel 60) are hydrophobic adsorbents, either with or without a functionalization*.

a *Specific surface area obtained from nitrogen sorption isotherms*.

b *Mean particle size obtained from SEM images*.

### Column Permeability and Packing Density

The back pressure was determined on an Agilent 1100 HPLC system through a glass column with a minimum column volume of 2 mL. The flow rates varied from 0.1 to 3 mL min^−1^ to assure the linear dependency of the pressure increase. The system pressure was observed with an empty column, and both frits pushed together. The specific adsorbent pressure Δ*p* was then calculated from the difference of the system with an empty column *p*_*system*_ and the total pressure with a packed column *p*_*system*+*adsorber*_. The mobile phase was deionized (DI) H_2_O and the temperature was held at 25°C. Here, the interstitial porosity ε, the dynamic viscosity η, the particle diameter *d*_*p*_, the linear velocity and the column length are considered:

$$\Delta p\;=\;\frac{1}{k_{0}}\;\frac{\eta \;u_{0}\;L_{column}}{d_{p}^{2}}$$

With the factor *k*_0_ being

$$k_{0}\;=\;\frac{\varepsilon^{3}}{150\;{(1-\;\varepsilon )}^{2}}$$

The permeability *B* gives the resistance over the bed length subject to the dynamic viscosity η, the interstitial velocity *u*_0_ and pressure difference:

$$B\;=\;\frac{\eta \;u_{0}\;L_{column}}{\Delta p}$$

The packing density was considered for the adsorbent dry weight. These were washed firstly three times with 100% methanol and then dried for 3 h at 80°C. The dry adsorbents were weighted and then suspended in DI H_2_O for the packing procedure. Here, the suspension was filled into the glass column (0.66 cm Ø) and compressed under a flow of ~9 cm min^−1^ DI H_2_O. The remaining suspension was again dried for 12 h at 80°C and weighted afterwards. Hence, the difference of initial adsorbent mass and the remaining mass results in the mass inside the column *m*_*adsorbent, dry*_. The column volume was determined geometrically. This leads to the packing density *p*:

$$p\;=\;\frac{m_{adsorbent,\;dry}}{V_{column}}$$

### HETP and Porosities

The plate number, the asymmetry factor and the porosities of the packed beds were determined experimentally with different tracer solutes, commonly used in reversed phase chromatography on an Agilent 1100 HPLC System with an UV/Vis detector. The column had a diameter of 0.66 cm and a total length of 15 cm. To determine the system dead volume correctly, the column was connected empty to the system and the frits were pushed together completely. The interstitial porosity was measured with blue dextran (Sigma-Aldrich, MW 2 MDa, CAS-Nr. 87915-38-6) and further measurements were conducted with uracil (Sigma-Aldrich, >99%, CAS-Nr. 66-22-8) for the total porosity. An injection volume of 2 μL was set for uracil (0.1 g L^−1^) and 10 μL for blue dextran (10 mM). Uracil and blue dextran were detected at a wavelength of 254 and 260 nm, respectively. All measurements were done as triplicates. The HETP was determined from the injection with uracil, as it has a similar size as β-caryophyllene and does not interact with the stationary phase. The calculation for the uracil specific plate number (*N*_*i*_) was done with the empirical equation for asymmetric peaks (Bidlingmeyer and Warren, 1984; Gritti and Guiochon, 2012):

$$N_{i}\;=41.7(\frac{{(\frac{t_{Ri}}{w_{0.1h}})}^{2}}{1.25\;+\;\frac{b_{0.1h}}{{\text{a}}_{0.1h}}})$$

Where *t*_*Ri*_ is the net retention time, *w*_0.1*h*_ the peak width at 10% height, *b*_0.1*h*_ and *a*_0.1*h*_ the width of the right and left half of *w*_0.1*h*_, respectively. The asymmetry factor is defined as $A_{S}=\;\frac{b_{0.1h}}{a_{0.1h}\;}$. If the peak is ideally symmetrical (*a*_0.1*h*_ = *b*_0.1*h*_), the equation reduces to:

$$N_{i}\;=\;5.54\;{\left(\frac{t_{Ri}}{w_{0.5h}}\right)}^{2}$$

Considering the column length *L*, the HETP can be calculated as follows:

$$HETP\;=\;\frac{L}{N}$$

The reduced HETP *h* considers the mean particle size *d*_*P*_ and can then be compared independently (Gritti and Guiochon, 2006):

$$h\;=\;\frac{HETP}{d_{p}}$$

A low HETP indicates an efficiently packed bed with a preferable mass transfer through the column. Here, only the HETP for uracil is compared throughout the investigated adsorbents and no adsorption of a solute is considered. Furthermore, 100% DI H_2_O was kept as the mobile phase because the adsorbents were loaded in an aqueous feed solution. It should be mentioned that for polymeric adsorbents a changing solvent concentration will lead to different degrees of swelling and therefore to changes in the bed composition (i.e., particle porosities).

With the measured retention volumes of uracil and blue dextran, the interstitial porosity ε is given as:

$$\varepsilon \;=\;\frac{V_{void}}{V_{column}}=\;\frac{V_{R,BlueDextran}}{V_{column}}$$

The total porosity ε_*t*_:

$$\varepsilon_{t}\;=\;\frac{V_{void}+\;V_{pore}}{V_{column}}=\;\frac{V_{R,Uracil}}{V_{column}}$$

From that, the particle porosity ε_*P*_ can be calculated:

$$\varepsilon_{p}=\;\frac{V_{pore}}{V_{ads}}=\;\frac{\varepsilon_{t}-\varepsilon }{1-\;\varepsilon }$$

### Batch Adsorption

Single adsorption isotherm data were determined with the common elution solvents acetonitrile and ethanol. Due to the strong hydrophobic interaction and extremely low solubility in water, a feasible measurement for the isotherms in pure water was not possible. Adsorption points were measured for three different solvent concentrations (60, 80, and 100%), and three different adsorbent concentrations (0.2, 0.6, and 1 g L^−1^). The concentrations of the solute and the solvent were selected in dependence of the maximum solubility. All measurements were conducted as triplicates (boundary values) or pentaplicates (middle values). The adsorbents were washed prior three times with pure methanol and further dried at 80°C for 24 h. Approximately 10 mg of dry adsorbent was then weighed on an analytical balance (XS205 DualRange, Mettler Toledo) into a 350 μL pointed glass insert with a PTFE cap. β-Caryophyllene was firstly dissolved in the solvent of choice (acetonitrile or ethanol) and then diluted into the glass inserts to a total volume of 200 μL. Controls without adsorbent were filled for a reliable quantification of the solutes. To avoid evaporation, the solutions were cooled on ice while pipetting. Afterwards, the filled glass vials were closed, inserted into a 96-well plate and centrifuged at 500 g for 1 min to remove air inside the adsorbent and bring the solution into complete contact. The plates were then incubated for 12 to 14 h at 25°C and 250 rpm. Then, the plates were centrifuged again at 500 g for 1 min and put into the HPLC and tempered at 25°C. 20 μL of the supernatant was injected for the quantitative analysis of β-caryophyllene. The controls without the adsorbent defined the initial concentration *c*_0_ and the supernatant concentration with the adsorbent gave the equilibrium concentration *c*_*eq*_. With the measured dry mass of the adsorbent *m*_*A*_ and an initial loading *q*_0_ of zero, the equilibrium load q for a defined equilibrium concentration can be calculated as follows:

$$\begin{array}{l} V_{R}\left(c_{0}\;-\;c_{eq}\right)\;=\;m_{A}\left(q\;-\;q_{0}\right)\;\to \;\\ \;q\;=\;\frac{V_{R}(c_{0}-\;c_{eq})}{m_{A}}+\;q_{0} \end{array}$$

Assuming a linear range at the lowest investigated concentration (0.2 g L^−1^), which is given at c < < k, the relationship for the adsorption reduces to *q*(*c*) = *H c*, with the Henry constant *H* (Schmidt-Traub et al., 2013). By this, the affinity at a certain condition can be evaluated and compared for all adsorbents.

### HPLC Analysis

The quantitative analysis of β-caryophyllene was conducted on an Agilent 1100 HPLC system with a UV/Vis detector. A reversed phase column (Kinetex, 5 μm EVO C18, 100 Å 150 × 3.0 mm, Phenomenex inc.) was installed. The mobile phases were DI H_2_O (solution A) and 90% acetonitrile (solution B). Both were acidified to 0.5% acetic acid and then filtered (0.22 μm) and degassed. β-Caryophyllene was analyzed in an isocratic mode with a flow rate of 0.5 mL min^−1^ and a column temperature of 40°C. A mobile phase ratio of 10:90 (A:B) was set and the samples were measured at 225 nm. The sample volume was set to 20 μL. A standard curve for β-caryophyllene from 0.01 to 1.2 g L^−1^ was determined beforehand. The evaluation was done on the ChemStation for LC 3D system software (Agilent Technologies).

### Retention Factors

Another method to determine the adsorption isotherm at a linear range is the measurement of the retention factor *k'* for small injection concentrations (c < < k) (Gritti et al., 2006; Guiochon et al., 2006). With the given phase ratio $\varphi =\frac{\left(1-\varepsilon_{t}\right)}{\varepsilon_{t}}$, the Henry constant defines as $H=\frac{k^{\prime }}{\varphi }$. The retention factor is determined as follows:

$${\text{k}}^{\prime }=\;\frac{t_{Ri}-\;t_{0}}{t_{0}}$$

Where *t*_*Ri*_ is the retention time of the solute and *t*_0_ the retention time of a non-interacting tracer (Gritti et al., 2007). This tracer should be the same size as the solute to assure the same fluid dynamical pathway through the column. The retention factors were measured in an isocratic mode in dependence of the solvent concentration (acetonitrile and ethanol) starting with 100% solvent and reducing the ratio in 5% (or 2%) steps. The packed beds were washed with 20 column volumes (CV) with the right concentration before the measurement. Measurements were stopped when the retention was too high for analysis.

### Frontal Analysis (FA) and Dynamic Binding Capacity (DBC)

Adsorption isotherms were measured for the Rensa^®^ RP system with different ethanol concentrations. Here, the frontal analysis (FA) method has been used for the isotherms (Gritti et al., 2003; Gritti and Guiochon, 2005b). It is one of the most precise but also elaborate methods for the determination of adsorption isotherms (Guiochon et al., 2006; Fornstedt, 2010). FA experiments were conducted on an ÄKTApurifier system and the detection was measured through UV/Vis at a wavelength of 210 nm. The system dead volume *V*_0_ was determined with the measurement of the breakthrough of the β-caryophyllene solution through the empty column, the frits were pushed together. The β-caryophyllene solution had a concentration of around 0.1 g L^−1^ with a defined ethanol concentration (70–100%). A washing solution contained the same ratio of ethanol for equilibrating the column. The breakthrough curve was measured for different concentrations *c*_*i*_ of the product solution (see Supplementary Figure 1) and fitted by a non-linear least square regression (see Supplementary Figure 2). After each run, the column was washed with 100% ethanol for 20 CV. The specific load *q(ci)* suspect to the concentration *c*_*i*_ and column volume V_column_ was calculated through the equivalent-area method and is defined as:

$$q(c_{i})\;=\;\frac{c_{i}\;(V_{eq}\;-\;V_{0})}{V_{column}}$$

The given loads were fitted empirically to various empirical isotherm models (see Supplementary Data), among the most common one, the Langmuir model:

$$q(c)\;=\;q_{\text{max}}\;\frac{k\;c}{1\;+\;k\;c}$$

With the maximum capacity *q*_*max*_ and the Langmuir constant *k*. A further method to compare the capacities under certain conditions is to determine the dynamic binding capacity (DBC). Mostly the increase of the breakthrough to 10% of the maximum concentration (DBC_10%_) is chosen and can be calculated as follows:

$$DBC_{10\%}\;=\;\frac{c_{0}\;\overset{.}{V}\;t_{10\%}}{V_{column}}$$

With the feed concentration *c*_0_, the flow rate $\overset{∙}{V}$, the net time until 10% breakthrough occurs t_10%_, and the column volume V_column_. The DBC curves were measured for β-caryophyllene at a concentration of 0.1 and 0.05 g L^−1^ (at 40%) and a flowrate of 1 mL min^−1^. The ethanol concentration was varied from 40 to 100%.

### Separation of β-Caryophyllene From *E. coli* Fermentation Mixture

A standard fermentation with sterilized complex media (Lysogeny Broth, LB, 0.1% pepton, 0.05% yeast extract, 0.1% NaCl) was done in a 1.5 L Bioreactor (BIOSTAT B plus, Sartorius, Göttingen, Germany) with the connected software BioPAT^®^ MFC/win. An *E. coli* W3110 strain (ATCC 27325) without antibiotics was cultivated to an optical density (λ = 600 nm) of around 40 by fed-batch cultivation and with glucose as a carbon source. The pH was controlled at 7 with ammonia (NH_3_). The broth was centrifuged at 3,000 g for 30 min and 1 L supernatant was filtered (0.22 μm). A standard solution of β-caryophyllene in methanol with a concentration of 3 g L^−1^ was given to 500 mL filtrated supernatant to a final concentration of ~10 mg L^−1^. The given solution was connected to an ÄKTApurifier system and pumped with a flow rate of 2 mL min^−1^ through a packed bed with Rensa^®^ RP (Ø = 0.66 cm, CV = 0.9 mL, HETP = 0.02 cm). The flow-through was collected in 10 mL fractions for offline β-caryophyllene analysis. After loading the column with the feed, gradient elution with an increase of 5% CV^−1^ was started and the elution fractions of 5 mL were collected. Further runs were conducted the same way, except for the gradient, which was adapted for a higher selectivity: A primary step at 50% ethanol was implemented and held for 10 CV as well as another step at 80% ethanol. The loading and the elution were monitored at the wavelengths 210 nm, 260 nm and 280 nm. β-caryophyllene quantification and purity analysis of the fractions were done on an Agilent 1100 HPLC system (see section HPLC analysis).

## Results

### Mechanical Characterization of Packed Bed Adsorbents

**Figure 2:**
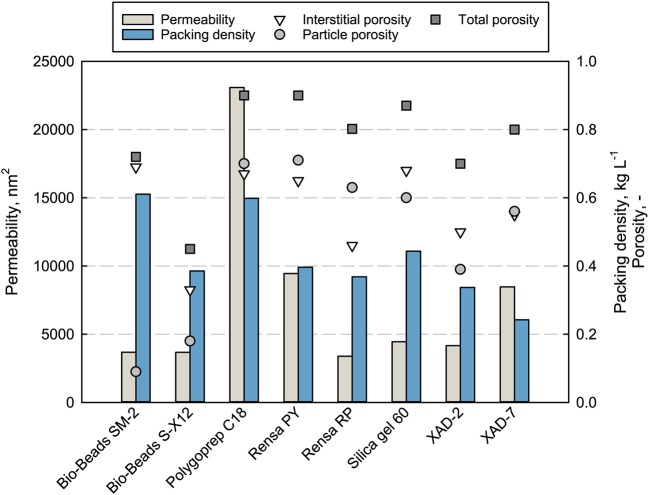
Properties (permeability, packing density, and total porosity) of the packed adsorbents in a chromatographic column, measured at 100% DI H_2_O with uracil and a flowrate of 1 mL min^−1^.

The main properties as particle size and cost per weight of the adsorbents are listed in Table 1. Mechanical stability and adsorbent properties are important parameters when it comes to production processes. A low permeability can limit the flow rate and therefore the productivity of the chromatographic process. The permeability of packed beds depends on the particle sizes, packing quality, porosities, and fluid characteristic as viscosity and flow rate. The measured permeability for every packed adsorbent as well as the packing density is shown in Figure 2 together with the determined total, interstitial and particle porosities (see Data Sheet 1).

The measured permeability does not correlate completely with the porosities or mean particle size. The silica-based adsorbent Polygoprep C18 shows significantly the highest permeability of 23,083 nm^2^ with the fourth biggest mean particle size as well as the highest total porosity of 0.9 together with the functionalized adsorbent Rensa^®^ PY. The calculated permeability (not shown) gives completely different values and orders for the adsorbents. Nevertheless, some tendencies can be seen, such as the lowest permeability for the smallest material, Rensa^®^ RP. Therefore, the mechanical description and calculation is not enough or correct for hydrophobic preparative adsorbents. This can be explained by two assumptions of the Darcy equation. First, a homogeneously packed bed and second, the sole presence of monodisperse and round particles. Both assumptions are not completely correct in the case of preparative adsorbents. A further assumption is that the surface resistance does play a role in an increasing back pressure. Consequently, the permeability must be determined experimentally for the process development.

Though the terpenes belong to the class of small molecules and pore diffusion limitation does not occur as significantly as in protein chromatography, the porosities of the packed bed and particles give a better insight in mass transport in chromatography. Porosities are essential parameters for mechanistic modeling of chromatographic processes. A high particle porosity correlates with a higher specific surface and therefore with more binding sites, which is preferable for small molecules. But the stability of the adsorbent decreases with increasing porosity and limits the process flow rate. The total porosity ε_*t*_ of the investigated packed beds lies in a range between 0.4 and 0.9, similar to the media described earlier by Schmidt-Traub et al. (2013). Besides that, the interstitial porosities ε vary between 0.33 and 0.69, where a mean value of 0.37 is assumed for round and monodisperse particles (Schmidt-Traub et al., 2013). The biggest differences can be observed for the particle porosities ε_*p*_, starting from 0.09 for Bio-Beads™ SM-2 and going to 0.71 for Rensa^®^ PY. Extremely low particle porosities can be explained by strong crosslinking of the polystyrene matrix that may lead to higher particle stabilities.

Packing density depends on the particle size distribution and the particle density itself. Bigger adsorbents as the XAD and Bio-Beads™ SM-2 show slightly lower densities between 0.25 and 0.4 g L^−1^. Silica gel 60 has a slightly higher density followed by the non-porous Bio-Beads™ S-X12 and Polygoprep C18 with 0.6 g L^−1^. Through the packing density data, a comparison between batch adsorption isotherms and in-column capacities is feasible.

**Figure 3:**
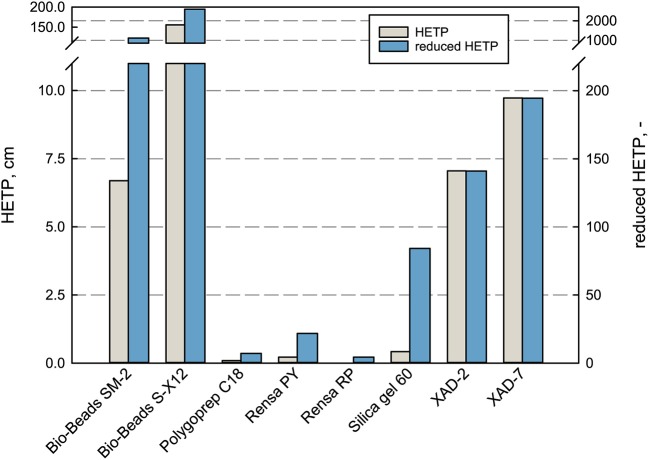
Performance characterization of the packed bed adsorbents in terms of HETP and reduced HETP. The values were determined at 100% DI H_2_O with uracil at 1 mL min^−1^ flow rate.

In terms of separation performance of a chromatographic column, the HETP is taken for evaluation and comparison. It depends on the plate number *N*, the parameter for column efficiency (Schmidt-Traub et al., 2013). A low HETP is preferred as the mass transport to and from the adsorbent surface is efficient and the concentration peak remains narrow (Gritti and Guiochon, 2010). By that, the product purity and concentration can be increased significantly with an efficient bed. In terms of the purification of terpenes, the efficiency plays a role in the adsorption phase and especially in the desorption phase, where an applied elution gradient can improve the purity and selectivity of the product. Therefore, the HETP and the reduced HETP *h* were measured and compared for every adsorbent in this work (see Figure 3).

The measured HETP values differ enormously for the investigated adsorbents. For the large-sized adsorbents Bio-Beads™ SM-2, XAD-2, and XAD-7 as well as the non-porous Bio-Beads™ S-X12, the theoretical plate height exceeded the here applied column length, which means that not even one equilibrium plate is reached within the bed. Whereas, the smaller materials lead to much higher efficiencies with HETP values ranging between 0.022 cm for Rensa^®^ RP and 0.42 cm for Silica gel 60. The reduced HETP *h* does indeed reduce the magnitude between the bigger and smaller particles but gives the same order of efficiency. These results give a profound basis for the later decision in process development when it comes to target values and possible leverages. The classical adsorbents are much cheaper but can be a disadvantage in the later process. Especially in the case of pharmaceutical ingredients and chemical precursors, the purity requirements are extremely high and must be met.

### Thermodynamic Characterization of the Adsorbents

**Figure 4:**
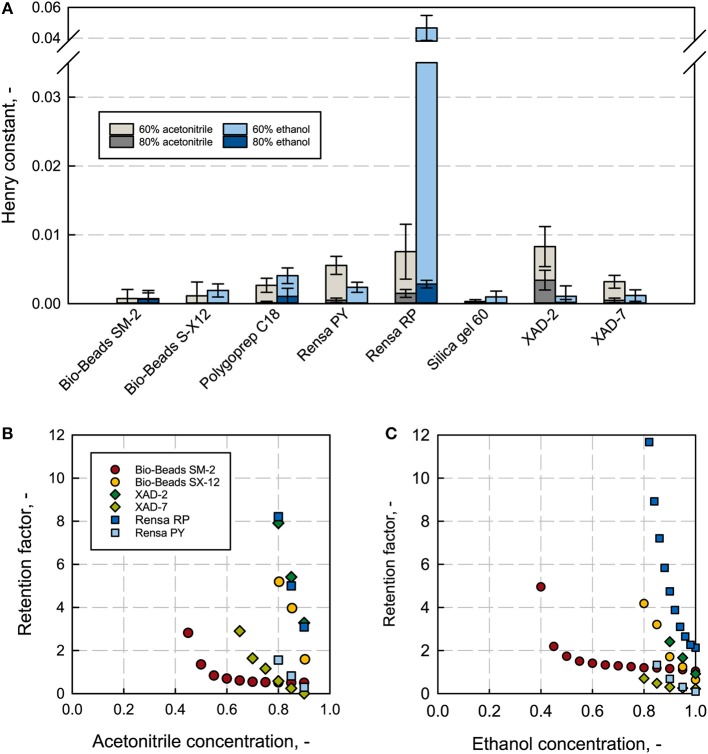
Henry constants from batch adsorption experiments at an initial concentration of 0.1 g L^−1^ β-caryophyllene **(A)** for all investigated adsorbents at different ethanol (blue) and acetonitrile (gray) concentrations. Rensa^®^ RP shows the highest Henry constant with 60% ethanol. Retention factors for different adsorbents with β-caryophyllene **(B,C)** subject to the solvent concentration of ethanol and acetonitrile show extreme variances in the affinity.

The Henry constant H describes the increase of the adsorption isotherm at a linear range; this indicates the affinity of a low concentrated solute, like the here discussed terpene (app. few 100 mg L^−1^), for the stationary phase. The higher the Henry constant the stronger the adsorption and thus, higher selectivities are given. Nevertheless, too strong binding can lead to an incomplete recovery due to the strong hydrophobic interactions on the column, leading to a necessarily appropriate selection of an elution solvent, with the required properties as water-miscibility and low toxicity. Both acetonitrile and ethanol are chemically diverse and can hence lead to different elution properties (Gritti and Guiochon, 2005a; Bocian et al., 2015; Saffarionpour et al., 2018b). The Henry constant was determined at different solvent concentrations. The Henry constants for 0 and 100% solvent were not plotted because at 100% solvent no binding was observed and at 0% (pure H_2_O) the binding was too strong and not quantifiable. The low solvent-concentration regime gives comparable values and shows the biggest differences in the measured affinity (see Data Sheet 5). For β-caryophyllene, the polystyrene adsorbent Rensa^®^ RP shows the highest H-values with 0.046 at 60% ethanol and the second highest with 0.0076 at 60% acetonitrile, right after XAD-2 with 0.0083 (see Figure 4A). The differences between the two solvents are significant. Rensa^®^ RP gives the highest Henry constant with a factor 10 higher than any other adsorbent at 60% ethanol. The H-values are directly comparable for the same type of solvent, solvent concentration and solute, and can give a reliable conclusion for the right adsorbent. The affinity in the linear range is furthermore a reliable indicator for a non-linear adsorption (Gritti and Guiochon, 2005b). By this, Rensa^®^ RP can be considered as the most suitable adsorbent for β-caryophyllene from the here investigated resins. The polar silica gel, which was taken for the negative test, showed the lowest affinities at all conditions. Compared to the Henry constant (calculated from batch experiments) the retention factors are determined at lower concentrations of the target molecule in the packed bed (Miyabe et al., 2001; Guiochon et al., 2006). The retention factor increases as well as the henry constant with a decreasing solvent concentration in reversed phase chromatography (see Figures 4B,C and Data Sheet 3). The measured high affinities of β-caryophyllene toward the polystyrene Rensa^®^ RP in Figure 4 can be confirmed with the determined retention factors. Here, the retention factor increases significantly with the decreasing ethanol and acetonitrile concentration and shows the steepest increase for all adsorbents. With acetonitrile as a solvent, XAD-2 shows a high retention, too, which can also be confirmed with the batch adsorption results.

The order of affinity compared to the order of retention factors are identical to each other except for one adsorbent, the almost non-porous Bio-Beads™ SM-2. This can be explained by the small specific surface (Gritti and Guiochon, 2007) (286 m^2^ g^−1^) and the high material density (0.6 kg L^−1^). In the batch adsorption experiment, a resin weight of only 10 mg was set and the specific density not considered. Hereby, a linear regime for the specific measurement with this adsorbent is excluded and a saturation phase is reached. In the case of the non-porous Bio-Beads™ S-X 12, though it has a specific surface of only 10 m^2^ g^−1^, a difference in order cannot be observed. This is possibly due to a general low affinity in the whole isotherm regime. Nevertheless, the other measured values seem to lie in the linear range and are comparable with β-caryophyllene as a solute. The retention factors are generally lower with ethanol than acetonitrile, resulting in a preferable higher desorption strength.

### Process for the Purification of β-Caryophyllene From *E. coli* Supernatant With Rensa^®^ RP and Ethanol

**Figure 5:**
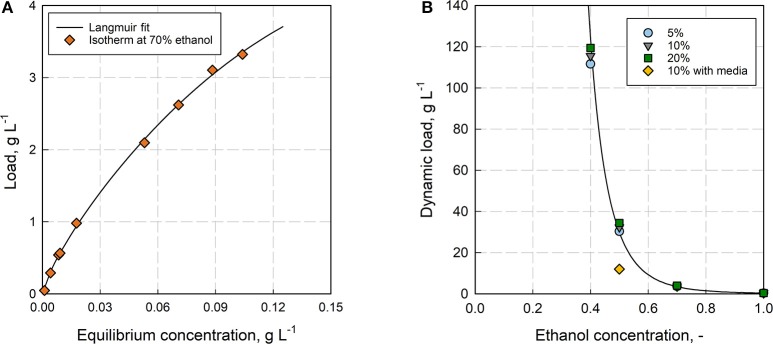
**(A)** Adsorption isotherm for β-caryophyllene on Rensa^®^ RP at 70% ethanol determined with the frontal analysis (FA). **(B)** Dependence of the Dynamic Binding Capacity (DBC) subject to the ethanol concentration for β-caryophyllene on Rensa^®^ RP. Shown are the capacities for the breakthrough at 5, 10, and 20% (relatively to the initial concentration; Column volume = 0.46 mL, flow rate = 2.9 cm min^−1^, HETP = 0.02 cm).

The adsorbent Rensa^®^ RP in combination with ethanol was chosen for the preparative separation of β-caryophyllene from aqueous fermentation mixtures. It gives the most efficient bed with the highest affinity toward the product. One trade-off is the low permeability, which limits the loading velocity. The first step in process development was the thorough determination of the adsorption isotherm and the dynamic binding capacity (see Figure 5A). The isotherm could not be determined in 100% H_2_O due to the low solubility and was therefore measured at 70% ethanol. Clearly, the isotherm shifts significantly with a changing ethanol concentration as it was already observed for polyphenols by Silva et al. (2018a), but the result gives a further insight into adsorption behavior of the solute. The isotherm was fitted with several empirical isotherm models (see Supplementary Figure 3 and Data Sheet 4), resulting in the best fit by the Langmuir model (*R*^2^ = 0.99). Besides the isotherm, the dynamic binding capacity was measured also in dependence of the ethanol concentration (see Figure 5B). Here, the dynamic load increases exponentially with a decreasing ethanol concentration. Further impact from the media components showed a significant decrease of the capacity with LB-media at 50% ethanol. The binding capacity (DBC_10%_) reached at this point 32.3 g L^−1^, whereas the capacity decreased to 12 g L^−1^ with LB-media instead of DI H_2_O. Comparing the dynamic binding capacity with the determined isotherm at 70% ethanol, an increase from DBC_10%_ = 3.6 g L^−1^ to q_max_ = 13.5 ± 4.0 g L^−1^ (95% confidence bound, Langmuir fit, see Supplementary Figure 4 and Data Sheet 2) can be observed.

The static capacity is always higher than the dynamic capacity at 10% breakthrough, due to the reduced residence time and the incomplete utilization of binding sites, which explains the more than twice the higher value. A maximum DBC_10%_ of 115.5 g L^−1^ was determined at 40% ethanol. Looking at the retention factors from Figure 4 and comparing them with the dynamic capacity, it can be clearly seen that there is just a tendency but not a clear correlation between the low concentration range (retention) and the saturated range (capacity) of the isotherm. The exponential increase of the retention factor from 100 to 80% ethanol does not result in a significant increase of the capacity at the same ethanol concentration. Instead, the exponential increase of the capacity is shifted toward much lower ethanol concentrations (<60%). Whereas, the retention factor gives the partitioning at low β-caryophyllene concentrations in dependence of ethanol, the capacity is affected by the multi-component adsorption of β-caryophyllene and ethanol. This is also the case for the adsorption with LB-media, where the capacity for β-caryophyllene decreased by over 70% because of impurity binding.

**Figure 6:**
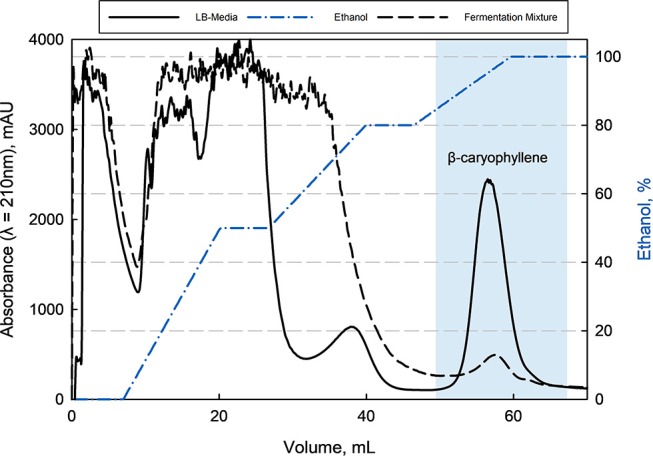
Elution chromatogram of β-caryophyllene from Rensa^®^ RP column (CV = 0.9 mL, HETP = 0.02 cm) after loading with LB-media or fermentation supernatant with an adapted ethanol gradient. The purity of >99% (HPLC) for β-caryophyllene could be reached by this single bind-and-elute step. The first major impurities are mostly peptides followed by a second impurity (fatty acids) at around 80% ethanol, which was separated by a factor >1.5 from the product.

With this information, a minimum capacity of 100 g L^−1^ in an aqueous solution was assumed for the preparative purification of β-caryophyllene from LB-media as well as from filtrated fermentation broth. Here was the loaded amount of β-caryophyllene 10 times lower than the determined capacity and a loss due to oversaturation was avoided. This can be confirmed by the offline analysis (HPLC) of the breakthrough. The following elution with ethanol of the adsorbed species showed a big fraction of impurities followed by β-caryophyllene at last (see Figure 6). The major impurities are hydrophobic peptides and HCP from the complex media and fermentation. Those can be separated through the adapted gradient until 80% ethanol. β-Caryophyllene elutes at 90% ethanol with a given separation factor of >1.5 toward the earlier impurities, which leads to a favored purity of >99% (HPLC). The elution yield was always > 80% but fluctuated extremely from batch to batch because of the small working concentration. A total productivity of the chromatographic process of 0.83 g β-caryophyllene h^−1^ L^−1^ was reached. At the same time a at least 40 times higher concentration of 0.4 g L^−1^ β-caryophyllene could be achieved after the ad- and desorption. A maximum loading velocity of 2.2 column volumes per minute was set. The amount of ethanol used per cycle is high with 10.8 L g^−1^ β-caryophyllene. A recovery cycle of the solvent should be therefore considered in an applied process to reduce costs. The resin usage of 0.225 L resin g^−1^ β-caryophyllene is acceptable and did not reach the maximum capacity.

## Conclusions

Our aim was to demonstrate the general applicability of the process chromatography for the purification of fermentatively produced terpenes as a serious and even better alternative to liquid-liquid extraction. Toxic and hazardous solvents can be avoided with the chromatographic isolation and higher purities can be achieved within one unit-operation. The crucial step in the chromatography is the selection of an appropriate adsorbent and solvent for elution. Adsorbents with the same chemical backbone can still vary in affinity toward a solute and the mechanical properties, namely the permeability and HETP, give a firm basis in material selection. With these factors and considering the frame given by the product (e.g., cost and purity requirements), the right adsorbent can be chosen rationally. Here, the polymeric material Rensa^®^ RP was selected after a thorough screening for the purification of the model terpene β-caryophyllene from a fermentation filtrate. It gave the highest efficiency of all investigated packed adsorbents (HETP = 0.022 cm) and the green and non-hazardous solvent ethanol was chosen for elution. With this system, a purity of >99% and a yield >80% were reached by a single chromatographic step. We demonstrated with the applied methods that the process chromatography is an alternative to the liquid-liquid extraction for microbially produced terpenes.

## Data Availability

All datasets generated for this study are included in the manuscript and/or Supplementary Files.

## Author Contributions

LG concepted and designed the work, analyzed and interpreted the data, and wrote the article. JK collected the data, analyzed and interpreted it, and approved the final version to be published. SB did the critical revision on the article and approved the final version to be published.

### Conflict of Interest Statement

The authors declare that the research was conducted in the absence of any commercial or financial relationships that could be construed as a potential conflict of interest.

## References

[B1] Ajikumar P. K. Xiao W.-H. Tyo K. E. J. Wang Y. Simeon F. Leonard E.. (2010). Isoprenoid pathway optimization for taxol precursor overproduction in *Escherichia coli*. Science 330, 70–74. 10.1126/science.1191652 20929806 PMC3034138

[B2] Awan A. R. Shaw W. M. Ellis T. (2016). Biosynthesis of therapeutic natural products using synthetic biology. Adv. Drug Deliv. Rev. 105, 96–106. 10.1016/j.addr.2016.04.010 27094795

[B3] Bergs D. Merz J. Delp A. Joehnck M. Martin G. Schembecker G. (2013). A standard procedure for the selection of solvents for natural plant extraction in the early stages of process development. Chem. Eng. Technol. 36, 1739–1748. 10.1002/ceat.201300276

[B4] Bhatti H. N. Khera R. A. (2013). Biotransformations of diterpenoids and triterpenoids: a review. J. Asian Nat. Prod. Res. 16, 70–104. 10.1080/10286020.2013.846908 24266458

[B5] Bidlingmeyer B. A. Warren F. V. (1984). Column efficiency measurement. Anal. Chem. 56, 1583A−1596A. 10.1021/ac00278a002

[B6] Biggs B. W. Lim C. G. Sagliani K. Shankar S. Stephanopoulos G. De Mey M.. (2016). Overcoming heterologous protein interdependency to optimize P450-mediated Taxol precursor synthesis in *Escherichia coli*. Proc. Natl. Acad. Sci. U.S.A. 113, 3209–3214. 10.1073/pnas.1515826113 26951651 PMC4812725

[B7] Bocian S. Soukup J. Jandera P. Buszewski B. (2015). Thermodynamics study of solvent adsorption on octadecyl-modified silica. Chromatographia 78, 21–30. 10.1007/s10337-014-2788-4 25568463 PMC4281355

[B8] Boghigian B. Salas D. Ajikumar P. Stephanopoulos G. Pfeifer B. (2012). Analysis of heterologous taxadiene production in K- and B-derived *Escherichia coli*. Appl. Microbiol. Biotechnol. 93, 1651–1661. 10.1007/s00253-011-3528-4 21850432 PMC9896015

[B9] Braga A. Silva M. Oliveira J. Silva A. R. Ferreira P. Ottens M. (2018). An adsorptive bioprocess for production and recovery of resveratrol with *Corynebacterium glutamicum*. J. Chem. Technol. Biotechnol. 93, 1661–1668. 10.1002/jctb.5538

[B10] Chang M. C. Keasling J. D. (2006). Production of isoprenoid pharmaceuticals by engineered microbes. Nat. Chem. Biol. 2, 674–681. 10.1038/nchembio836 17108985

[B11] Choi H.-K. Yun J.-H. Kim S.-I. Son J.-S. Kim H.-R. Kim J.-H. (2001). Enhanced production of paclitaxel by semi-continuous batch process (SCBP) in suspension culture of Taxus chinensis. Enzyme Microbial Technol. 29, 583–586. 10.1016/S0141-0229(01)00427-6

[B12] Dziggel C. Schäfer H. Wink M. (2017). Tools of pathway reconstruction and production of economically relevant plant secondary metabolites in recombinant microorganisms. Biotechnol. J. 12:1600145. 10.1002/biot.201600145 28009095

[B13] Felinger A. Guiochon G. (1998). Comparing the optimum performance of the different modes of preparative liquid chromatography. J. Chromatogr. A 796, 59–74. 10.1016/S0021-9673(97)01075-3 9513282

[B14] Fornstedt T. (2010). Characterization of adsorption processes in analytical liquid-solid chromatography. J. Chromatogr. A. 1217, 792–812. 10.1016/j.chroma.2009.12.044 20053406

[B15] Frense D. (2007). Taxanes: perspectives for biotechnological production. Appl. Microbiol. Biotechnol. 73, 1233–1240. 10.1007/s00253-006-0711-0 17124581

[B16] Gailliot F. P. Gleason C. Wilson J. J. Zwarick J. (1990). Fluidized bed adsorption for whole broth extraction. Biotechnol. Prog. 6, 370–375. 10.1021/bp00005a009 1366874

[B17] Gritti F. Felinger A. Guiochon G. (2006). Influence of the errors made in the measurement of the extra-column volume on the accuracies of estimates of the column efficiency and the mass transfer kinetics parameters. J. Chromatogr. A 1136, 57–72. 10.1016/j.chroma.2006.09.074 17046003

[B18] Gritti F. Gotmar G. Stanley B. J. Guiochon G. (2003). Determination of single component isotherms and affinity energy distribution by chromatography. J. Chromatogr. A, 988, 185–203. 10.1016/S0021-9673(02)02084-8 12641156

[B19] Gritti F. Guiochon G. (2005a). Adsorption mechanism in RPLC. Effect of the nature of the organic modifier. Anal. Chem. 77, 4257–4272. 10.1021/ac0580058 15987135

[B20] Gritti F. Guiochon G. (2005b). Critical contribution of nonlinear chromatography to the understanding of retention mechanism in reversed-phase liquid chromatography. J. Chromatogr. A 1099, 1–42. 10.1016/j.chroma.2005.09.082 16271269

[B21] Gritti F. Guiochon G. (2006). General HETP equation for the study of mass-transfer mechanisms in RPLC. Anal. Chem. 78, 5329–5347. 10.1021/ac060203r 16878867

[B22] Gritti F. Guiochon G. (2007). Comparison between the loading capacities of columns packed with partially and totally porous fine particles: what is the effective surface area available for adsorption? J. Chromatogr. A 1176, 107–122. 10.1016/j.chroma.2007.10.076 18001756

[B23] Gritti F. Guiochon G. (2010). A protocol for the measurement of all the parameters of the mass transfer kinetics in columns used in liquid chromatography. J. Chromatogr. A 1217, 5137–5151. 10.1016/j.chroma.2010.06.016 20609443

[B24] Gritti F. Guiochon G. (2012). Mass transfer kinetics, band broadening and column efficiency. J. Chromatogr. A 1221, 2–40. 10.1016/j.chroma.2011.04.058 21664619

[B25] Gritti F. Kazakevich Y. Guiochon G. (2007). Measurement of hold-up volumes in reverse-phase liquid chromatography: Definition and comparison between static and dynamic methods. J. Chromatogr. A 1161, 157–169. 10.1016/j.chroma.2007.05.102 17610882

[B26] Guerra-Bubb J. Croteau R. Williams R. M. (2012). The early stages of taxol biosynthesis: an interim report on the synthesis and identification of early pathway metabolites. Nat. Prod. Rep. 29, 683–696. 10.1039/c2np20021j 22547034 PMC3373433

[B27] Guiochon G. (2002). Preparative liquid chromatography. J. Chromatogr. A 965, 129–161. 10.1016/S0021-9673(01)01471-6 12236522

[B28] Guiochon G. Felinger A. Shirazi D. G. (2006). Fundamentals of Preparative and Nonlinear Chromatography, 2nd Edn. San Diego, CA: Elsevier. 10.1016/j.chroma.2016.03.050 27079748

[B29] Howat S. Park B. Oh I. S. Jin Y.-W. Lee E.-K. Loake G. J. (2014). Paclitaxel: biosynthesis, production and future prospects. New Biotechnol. 31, 242–245. 10.1016/j.nbt.2014.02.010 24614567

[B30] Huang Q. Roessner C. A. Croteau R. Scott A. I. (2001). Engineering Escherichia coli for the synthesis of taxadiene, a key intermediate in the biosynthesis of taxol. Bioorg. Med. Chem. 9, 2237–2242. 10.1016/S0968-0896(01)00072-4 11553461

[B31] Janoschek L. Grozdev L. Berensmeier S. (2018). Membrane-assisted extraction of monoterpenes: from *in silico* solvent screening towards biotechnological process application. R. Soc. Open Sci. 5:172004. 10.1098/rsos.172004 29765654 PMC5936919

[B32] Jennewein S. Croteau R. (2001). Taxol: biosynthesis, molecular genetics, and biotechnological applications. Appl. Microbiol. Biotechnol. 57, 13–19. 10.1007/s002530100757 11693909

[B33] Jiang M. Stephanopoulos G. Pfeifer B. (2012). Downstream reactions and engineering in the microbially reconstituted pathway for Taxol. Appl. Microbiol. Biotechnol. 94, 841–849. 10.1007/s00253-012-4016-1 22460591 PMC9896016

[B34] Kawasaki J. Kosuge H. Habaki H. Morita Y. (2008). Separation of taxane compounds by liquid-liquid extraction. Chem. Eng. Commun. 195, 644–660. 10.1080/00986440701555456

[B35] Keasling J. D. Mendoza A. Baran P. S. (2012). A constructive debate. Nature 492, 188–189. 10.1038/492188a 23235869

[B36] Krings U. Kelch M. Berger R. G. (1993). Adsorbents for the recovery of aroma compounds in fermentation processes. J. Chem. Technol. Biotechnol. 58, 293–299. 10.1002/jctb.280580314

[B37] Leavell M. D. McPhee D. J. Paddon C. J. (2016). Developing fermentative terpenoid production for commercial usage. Curr. Opin. Biotechnol. 37, 114–119. 10.1016/j.copbio.2015.10.007 26723008

[B38] Li Y. Zhang G. Pfeifer B. A. (2015). Current and emerging options for taxol production, in Biotechnology of Isoprenoids, eds Schrader J. Bohlmann J (Cham: Springer International Publishing),. 405–425. 10.1007/10_2014_292 25528175

[B39] Liu W. C. Gong T. Zhu P. (2016). Advances in exploring alternative Taxol sources. RSC Adv. 6, 48800–48809. 10.1039/C6RA06640B

[B40] Malik S. Cusidó R. M. Mirjalili M. H. Moyano E. Palazón J. Bonfill M. (2011). Production of the anticancer drug taxol in *Taxus baccata* suspension cultures: a review. Process Biochem. 46, 23–34. 10.1016/j.procbio.2010.09.004

[B41] Martin V. J. J. Pitera D. J. Withers S. T. Newman J. D. Keasling J. D. (2003). Engineering a mevalonate pathway in *Escherichia coli* for production of terpenoids. Nat. Biotechnol. 21, 796–802. 10.1038/nbt833 12778056

[B42] McPartland T. J. Patil R. A. Malone M. F. Roberts S. C. (2012). Liquid-liquid extraction for recovery of paclitaxel from plant cell culture: solvent evaluation and use of extractants for partitioning and selectivity. Biotechnol. Prog. 28, 990–997. 10.1002/btpr.1562 22581674 PMC3418474

[B43] Miyabe K. Sotoura S. Guiochon G. (2001). Retention and mass transfer characteristics in reversed-phase liquid chromatography using a tetrahydrofuran–water solution as the mobile phase. J. Chromatogr. A 919, 231–244. 10.1016/S0021-9673(01)00821-4 11442028

[B44] Newman J. D. Marshall J. Chang M. Nowroozi F. Paradise E. Pitera D.. (2006). High-level production of amorpha-4,11-diene in a two-phase partitioning bioreactor of metabolically engineered *Escherichia coli*. Biotechnol. Bioeng. 95, 684–691. 10.1002/bit.21017 16878333

[B45] Ongley S. E. Bian X. Neilan B. A. Muller R. (2013). Recent advances in the heterologous expression of microbial natural product biosynthetic pathways. Nat. Prod. Rep. 30, 1121–1138. 10.1039/c3np70034h 23832108

[B46] Paddon C. J. Westfall P. J. Pitera D. J. Benjamin K. Fisher K. McPhee D.. (2013). High-level semi-synthetic production of the potent antimalarial artemisinin. Nature 496, 528–532. 10.1038/nature12051 23575629

[B47] Ro D.-K. Paradise E. M. Ouellet M. Fisher K. J. Newman K. L. Ndungu J. M.. (2006). Production of the antimalarial drug precursor artemisinic acid in engineered yeast. Nature 440, 940–943. 10.1038/nature04640 16612385

[B48] Rohena C. C. Mooberry S. L. (2014). Recent progress with microtubule stabilizers: new compounds, binding modes and cellular activities. Nat. Prod. Rep. 31, 335–355. 10.1039/C3NP70092E 24481420 PMC4167679

[B49] Saffarionpour S. de Jong T. F. Van der Wielen L. A. M. Brouwer E. Ottens M. (2018a). Column chromatography for separation and fractionation of flavor-active esters on hydrophobic resins and simulation of breakthrough behavior. Separat. Purific. Technol. 210, 304–319. 10.1016/j.seppur.2018.05.008

[B50] Saffarionpour S. Tam S.-Y. S. Van der Wielen L. A. M. Brouwer E. Ottens M. (2018b). Influence of ethanol and temperature on adsorption of flavor-active esters on hydrophobic resins. Separat. Purific. Technol. 210, 219–230. 10.1016/j.seppur.2018.05.026

[B51] Schmidt-Traub H. Schulte M. Seidel-Morgenstern A. (2013). Preparative Chromatography, 2nd Edn. Weinheim: Wiley 10.1002/9783527649280

[B52] Silva M. Castellanos L. Ottens M. (2018a). Capture and purification of polyphenols using functionalized hydrophobic resins. Indus. Eng. Chem. Res. 57, 5359–5369. 10.1021/acs.iecr.7b05071 29755200 PMC5939897

[B53] Silva M. García J. C. Ottens M. (2018b). Polyphenol liquid–liquid extraction process development using NRTL-SAC. Indus. Eng. Chem. Res. 57, 9210–9221. 10.1021/acs.iecr.8b00613 30270975 PMC6156102

[B54] Soliman S. Tang Y. (2015). Natural and engineered production of taxadiene with taxadiene synthase. Biotechnol. Bioeng. 112, 229–235. 10.1002/bit.25468 25257933

[B55] Song M. C. Kim E. J. Kim E. Rathwell K. Nam S. J. Yoon Y. J. (2014). Microbial biosynthesis of medicinally important plant secondary metabolites. Nat. Prod. Rep. 31, 1497–1509. 10.1039/C4NP00057A 25072622

[B56] Sreekanth D. Syed A. Sarkar S. Sarkar D. Santhakumari B. Ahmad A. (2009). Production, purification, and characterization of taxol and 10-DABIII from a new Endophytic Fungus *Gliocladium* sp Isolated from the Indian Yew Tree, Taxus baccata. J. Microbiol. Biotechnol. 19, 1342–1347. 10.4014/jmb.0904.4041 19996685

[B57] Straathof A. J. J. (ed.). (2011). 2.57 - the proportion of downstream costs in fermentative production processes A2 - Moo-Young, Murray, in Comprehensive Biotechnology, 2nd Edn. (Burlington: Academic Press), 811–814. 10.1016/B978-0-08-088504-9.00492-X

[B58] Sun R. Fu K. Fu Y. Zu Y. Wang Y. Luo M.. (2009). Preparative separation and enrichment of four taxoids from Taxus chinensis needles extracts by macroporous resin column chromatography. J. Separat. Sci. 32, 1284–1293. 10.1002/jssc.200800689 19360728

[B59] Tsuruta H. Paddon C. J. Eng D. Lenihan J. R. Horning T. Anthony L. C.. (2009). High-level production of amorpha-4,11-Diene, a precursor of the antimalarial agent artemisinin, in *Escherichia coli*. PLoS ONE 4:e4489. 10.1371/journal.pone.0004489 19221601 PMC2637983

[B60] Ward V. C. A. Chatzivasileiou A. O. Stephanopoulos G. (2018). Metabolic engineering of *Escherichia coli* for the production of isoprenoids. Fems Microbiol. Lett. 365:fny079. 10.1093/femsle/fny079 29718190

[B61] Wen Y. Du H. Tu Y. Luo W. Li Q. Zhu C. (2015). Preparative enrichment and purification of nevadensin fromLysionotus pauciflorususing macroporous resins. Sep. Sci. Technol. 51, 339–347. 10.1080/01496395.2015.1085066

[B62] Winkelnkemper T. Schembecker G. (2010). Purification performance index and separation cost indicator for experimentally based systematic downstream process development. Separat. Purific. Technol. 72, 34–39. 10.1016/j.seppur.2009.12.025

[B63] Winkelnkemper T. Schuldt S. Schembecker G. (2011). Systematic downstream process development for purification of baccatin III with key performance indicators. Separat. Purific. Technol. 77, 355–366. 10.1016/j.seppur.2011.01.004

[B64] Xiong N. Yu R. Chen T. Xue Y.-P. Liu Z.-Q. Zheng Y.-G. (2019). Separation and purification of l-methionine from *E. coli* fermentation broth by macroporous resin chromatography. J. Chromatogr. B 1110–1111, 108–115. 10.1016/j.jchromb.2019.02.016 30798071

[B65] Yamada Y. Kuzuyama T. Komatsu M. Shin-ya K. Omura S. Cane D. E.. (2014). Terpene synthases are widely distributed in bacteria. Proc. Natl. Acad. Sci. U.S.A. 112, 857–862. 10.1073/pnas.1422108112 25535391 PMC4311827

