# Supplementary material for: One-Step Purification of Microbially Produced Hydrophobic Terpenes via Process Chromatography
Source: Front Bioeng Biotechnol. 2019 Jul 29;7:185. doi: 10.3389/fbioe.2019.00185 (PMC6681792; doi:10.3389/fbioe.2019.00185)
Supplement: Supplementary file 1 [file Presentation_1.pdf]

Supplementary Material

1 Supplementary Data

Empirical fits for adsorption isotherms:

Variables for the empirical isothermic fits described in this work:

qmax	Maximum capacity (mg mL^{-1})
k	Equilibrium constant
x	Concentration of solute in bulk phase in equilibrium
n	Empirical parameter

Langmuir: $qmax\ \frac{x \cdot k}{1+(x \cdot k)}$

Langmuir-Freundlich: $qmax\ \frac{(k \cdot x)^n}{1+(k \cdot x)^2}$

Toth: $qmax\ \frac{k \cdot x}{(1+(k \cdot x)^n)^{\frac{1}{n}}}$

Freundlich: $k \cdot x^{\frac{1}{n}}$

Jovanovic: $qmax\ (1 - \mathrm{e}^{-k \cdot x})$

Bi-Langmuir: $qmax_1\ \frac{k_1 \cdot x}{1+k_1 \cdot x} + qmax_2\ \frac{k_2 \cdot x}{1+k_2 \cdot x}$

2 Supplementary Figures and Tables

2.1 Supplementary Figures

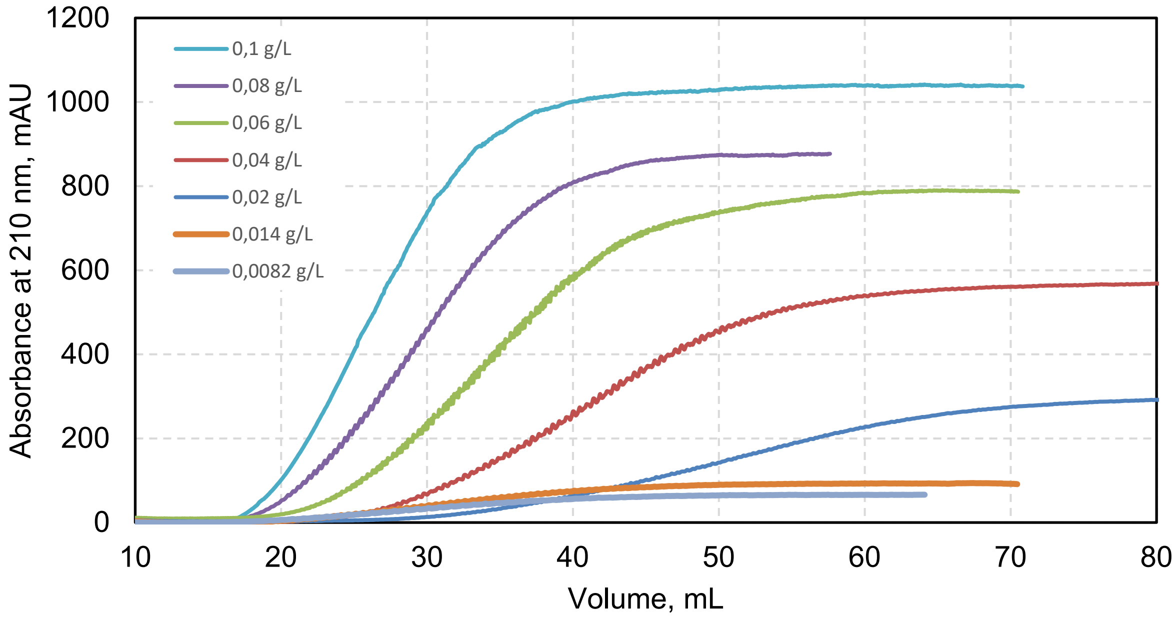

Supplementary Figure 1: Measured breakthrough curves for the determination of the adsorption isotherms of caryophyllene on Rensa RP®.

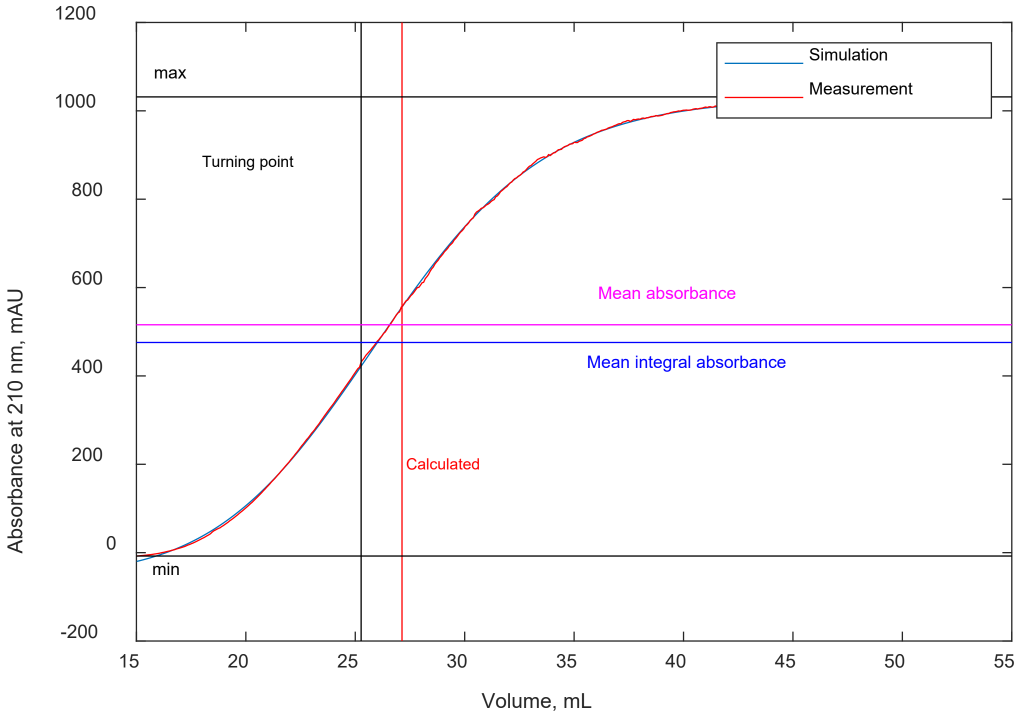

Supplementary Figure 2: Graphical result of the determined isothermic point as described by Gritti1. It is the point, where the integral under the absorbance until the calculated point equals the integral of the area between the maximum value and the absorbance starting from the calculated point. It must not be mistaken with the mean integral absorbance or the mean absorbance (as shown).

1. Gritti, F.; Gotmar, G.; Stanley, B. J.; Guiochon, G., Determination of single component isotherms and affinity energy distribution by chromatography. *Journal of Chromatography A* **2003,** *988* (2), 185-203.

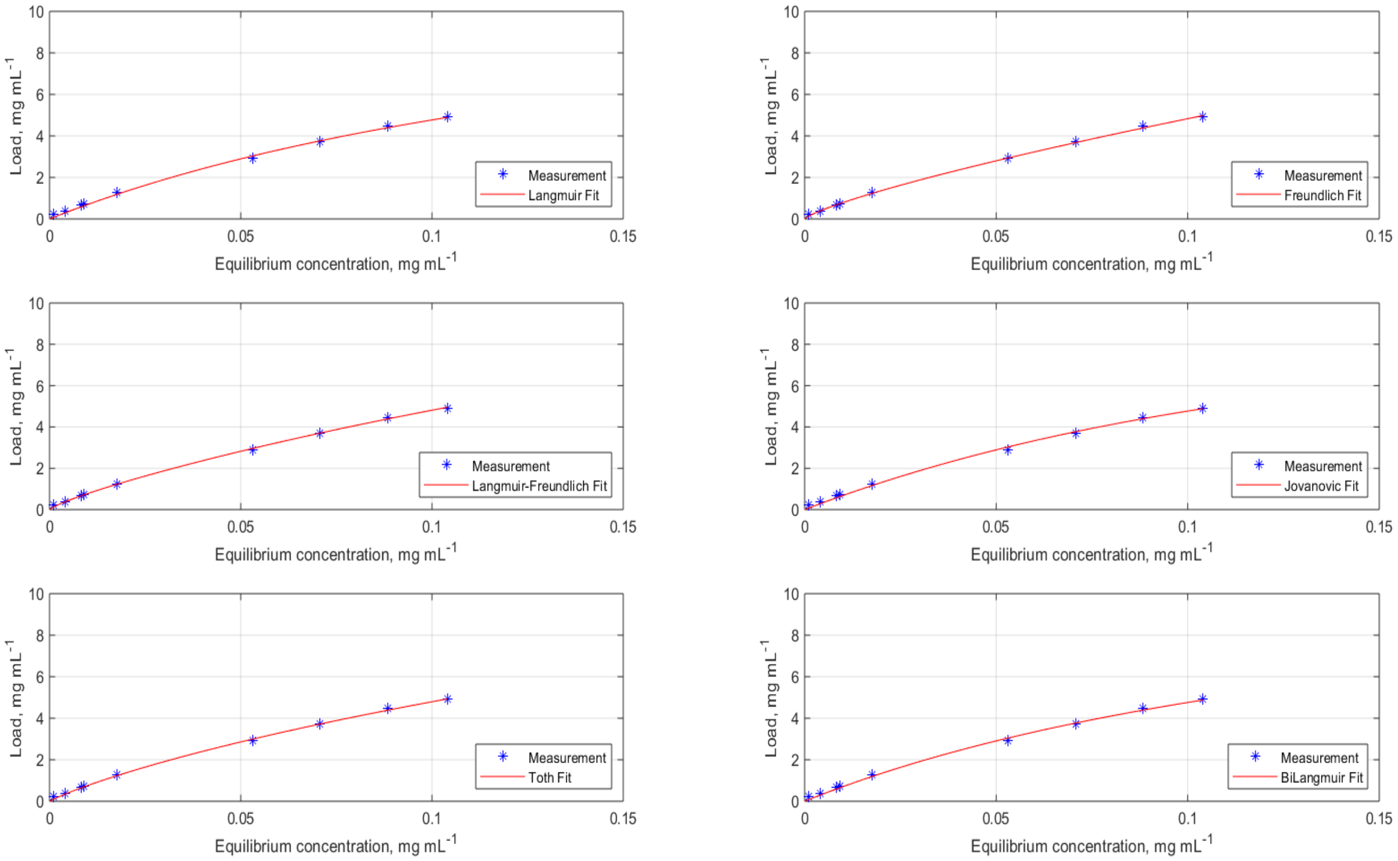

Supplementary Figure 3: Various empirical fits for the isotherm of β-caryophyllene on Rensa RP® with 70% ethanol. The models used were Langmuir, Freundlich, Langmuir-Freundlich, Jovanovic, Toth and BiLangmuir.

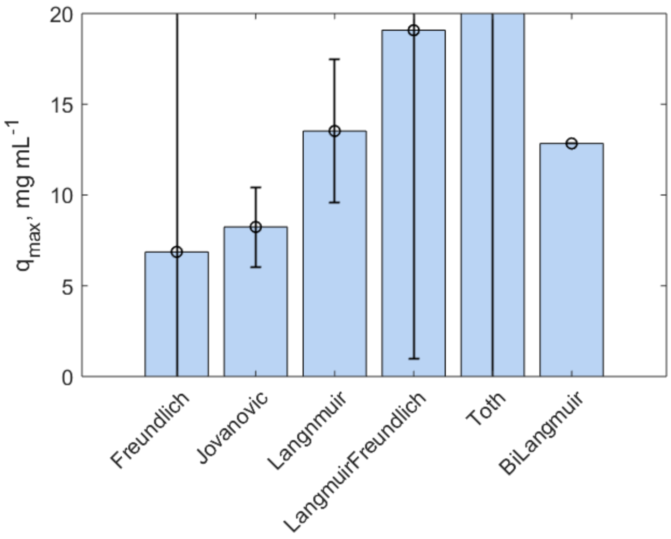

Supplementary Figure 4: Calculated maximum capacity qmax for β-caryophyllene on Rensa RP with 70% ethanol for the given empirical isothermic models.
